# Inhibitory mechanisms of docosahexaenoic acid on carbachol-, angiotensin II-, and bradykinin-induced contractions in guinea pig gastric fundus smooth muscle

**DOI:** 10.1038/s41598-024-62578-y

**Published:** 2024-05-22

**Authors:** Keyue Xu, Miyuki Shimizu, Toma Yamashita, Mako Fujiwara, Shunya Oikawa, Guanghan Ou, Naho Takazakura, Taichi Kusakabe, Keisuke Takahashi, Keisuke Kato, Kento Yoshioka, Keisuke Obara, Yoshio Tanaka

**Affiliations:** 1 Department of Chemical Pharmacology, Faculty of Pharmaceutical Sciences, Toho University, Miyama 2-2-1, Funabashi-City, Chiba 274-8510 Japan; 2 Department of Organic Chemistry, Faculty of Pharmaceutical Sciences, Toho University, Miyama 2-2-1, Funabashi-City, Chiba 274-8510 Japan

**Keywords:** Stomach, Fatty acids

## Abstract

We studied the inhibitory actions of docosahexaenoic acid (DHA) on the contractions induced by carbachol (CCh), angiotensin II (Ang II), and bradykinin (BK) in guinea pig (GP) gastric fundus smooth muscle (GFSM), particularly focusing on the possible inhibition of store-operated Ca^2+^ channels (SOCCs). DHA significantly suppressed the contractions induced by CCh, Ang II, and BK; the inhibition of BK-induced contractions was the strongest. Although all contractions were greatly dependent on external Ca^2+^, more than 80% of BK-induced contractions remained even in the presence of verapamil, a voltage-dependent Ca^2+^ channel inhibitor. BK-induced contractions in the presence of verapamil were not suppressed by LOE-908 (a receptor-operated Ca^2+^ channel (ROCC) inhibitor) but were suppressed by SKF-96365 (an SOCC and ROCC inhibitor). BK-induced contractions in the presence of verapamil plus LOE-908 were strongly inhibited by DHA. Furthermore, DHA inhibited GFSM contractions induced by cyclopiazonic acid (CPA) in the presence of verapamil plus LOE-908 and inhibited the intracellular Ca^2+^ increase due to Ca^2+^ addition in CPA-treated 293T cells. These findings indicate that Ca^2+^ influx through SOCCs plays a crucial role in BK-induced contraction in GP GFSM and that this inhibition by DHA is a new mechanism by which this fatty acid inhibits GFSM contractions.

## Introduction

Docosahexaenoic acid (DHA), abundant in fish oil, has been shown to prevent various cardiovascular diseases and to exhibit beneficial effects on other conditions such as inflammatory diseases, neurodegenerative diseases, malignant tumors, autoimmune diseases, diabetes, and dyslipidemia^1–5^. Some of the preventive/beneficial effects of the long-term intake of DHA are generally assumed to be related to the suppression of inflammatory prostanoid production^6^. However, we previously showed that DHA immediately and selectively inhibited the contractions induced by U46619 (a thromboxane A_2_ (TXA_2_) mimetic) and prostaglandin F_2α_ (PGF_2α_) in vascular and tracheal smooth muscles (SMs)^7–11^. Furthermore, based on the results of studies with human prostanoid TP receptor-expressing cells, we demonstrated that an antagonistic effect against TP receptors is partly responsible for the immediate inhibitory actions exhibited by DHA^10^. Binding studies with human platelets also showed an antagonistic action of DHA versus TP receptor ligands with a p*K*_i_ value of 5.24^12,13^.

TP receptors play an important role in the contractile regulations of not only tonic SMs including blood vessel and tracheal SMs, but also phasic SMs including gastrointestinal tract SM^14–16^. For instance, we reported that TP receptors play a significant role in the contractions induced by U46619 and some prostanoids in gastric fundus (GF) SM (GFSM) in guinea pig (GP)^17^. Against these contractions, we showed that DHA exhibited inhibitory activity that was partly attributed to its competitive antagonism versus TP receptors; the p*A*_*2*_ value of DHA versus U46619 was calculated to be 5.13^17^, which was equivalent to the value of 5.16 obtained in porcine coronary artery^10^. Functional inhibition of voltage-dependent Ca^2+^ channels (VDCCs) was also assumed to be partly responsible for the DHA-induced suppression of GFSM contractions by U46619 and prostanoids^17^. Furthermore, the functional inhibition of VDCCs was shown to be involved in the DHA inhibitory actions on GP ileal/colonic longitudinal SM contractions induced by some prostanoids^18^.

In addition to prostanoids, non-prostanoid substances including acetylcholine, angiotensin II (Ang II), and bradykinin (BK) can be contractile regulators of GFSM^19–21^. Acetylcholine is involved in physiological contractions of GFSM, and its release from parasympathetic nerve endings causes gastric peristalsis^22^. Although fluctuations in plasma concentrations of Ang II in the physiological range are suggested not to play an important role in the normal regulation of gastric motility, angiotensin-positive neurons have also been shown to exist in the stomach and may play some roles locally^23^. Furthermore, in streptozotocin (STZ)-induced diabetic mice, expression of the Ang II AT_1_ receptor in GFSM and that of angiotensin converting enzyme (ACE) in gastric mucosa are reported to increase, and Ang II-induced GFSM contractions are greater in the diabetic mice than in normal mice^24^. Additionally, BK-induced GFSM contractions are reported to be enhanced in STZ-induced diabetic mice, which may be involved in the development or maintenance of the diabetic lesions^21^.

Regarding contractions induced by non-prostanoid biological substances such as acetylcholine and histamine, we previously showed that DHA partly inhibited their contractile effects on GP ileal/colonic longitudinal SMs by functionally inhibiting VDCCs^18^. We assumed that DHA would exhibit the same inhibitory actions against non-prostanoid substance-induced contractions even in GFSM. Therefore, this study was performed to test this hypothesis. Herein, we show the inhibitory actions of DHA against GP GFSM contractions induced by non-prostanoid substances, focusing on carbachol (CCh, a choline ester), Ang II, and BK, which were found to produce detectable and quantifiable contractions. In addition, we report evidence to support that store-operated Ca^2+^ channels (SOCCs) are also a target of DHA especially in its inhibitory actions against BK-induced contraction of GP GFSM.

## Results

### Inhibitory actions of DHA on GFSM contractions induced by CCh, Ang II, and BK

In the present study, we examined whether DHA inhibited contractions induced by non-prostanoid chemical stimulants. We focused on CCh, Ang II, and BK because, among the 22 chemicals tested, (1) sufficiently detectable and quantifiable contractile effects were observed with CCh, Ang II, and BK; (2) strong contractions were also induced by neurokinin A but could not be quantified; and (3) the other 18 biological substances (histamine, substance P, serotonin, melatonin, dopamine, glucagon-like peptide-1, urotensin II, atrial natriuretic peptide, neuromedin B, neuromedin C, neuromedin U, orexin A, motilin, galanin, guanosine, guanosine triphosphate, adenosine, and adenosine triphosphate) did not induce substantial contractions (data not shown).

**Figure 1:**
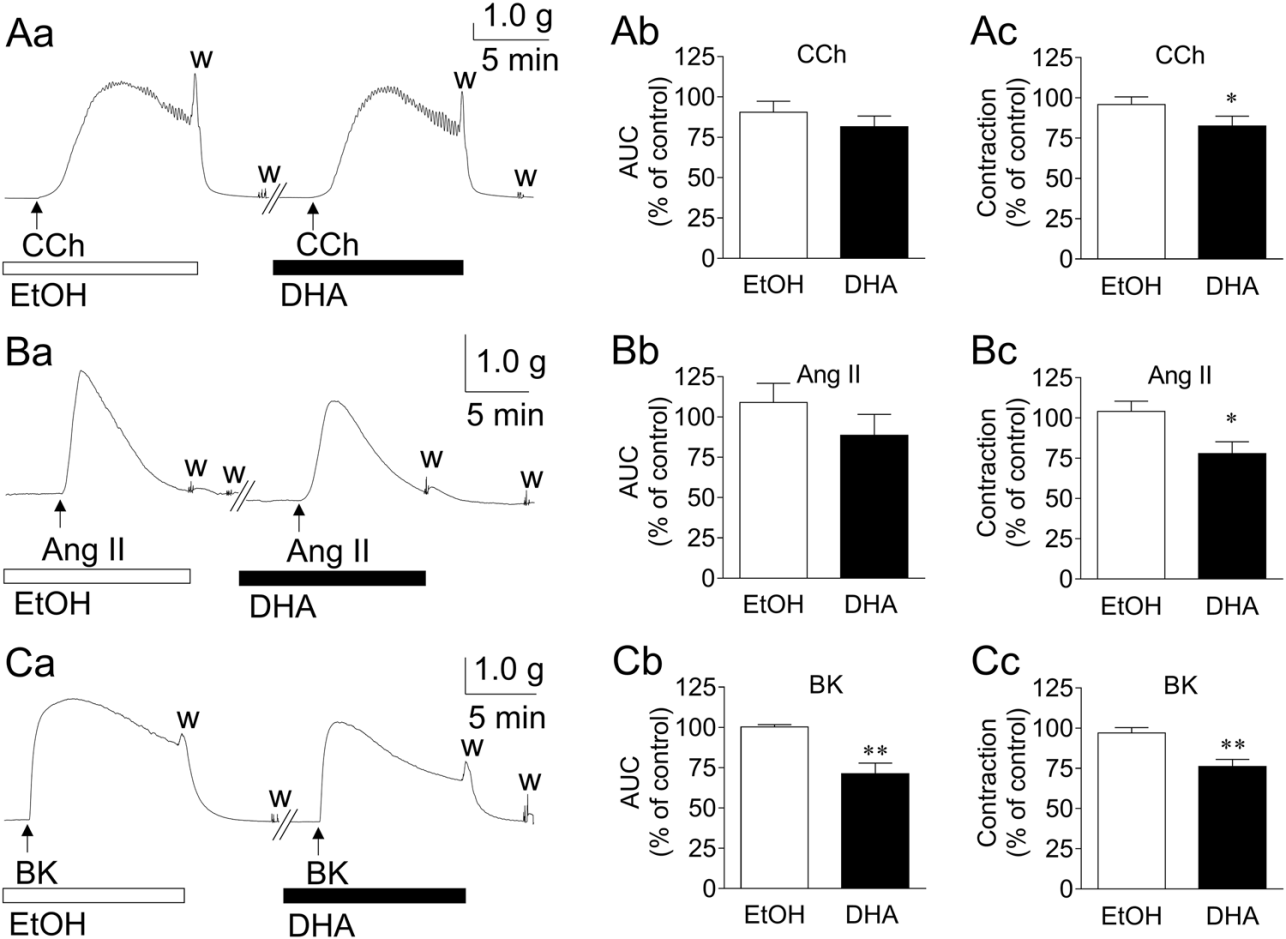
Representative traces (**a**) and summarized data (**b**: area under the curve (AUC); **c**: maximum contraction) of the inhibitory actions of docosahexaenoic acid (DHA, 3 × 10^−5^ M) on the contractions induced by carbachol (CCh, 6 × 10^−8^ M; **A**), angiotensin II (Ang II, 10^−7^ M; **B**), and bradykinin (BK, 10^−6^ M; **C**) in guinea pig gastric fundus smooth muscle. Data are expressed as means ± standard error of the mean (*n* = 6 (**A**, **B**), *n* = 5 (**C**)). *P < 0.05, **P < 0.01 versus EtOH (paired *t*-test). EtOH, ethanol (0.1%), w, wash out.

Figure 1 depicts the representative traces (a) and summarized data (b: area under the curve (AUC); c: maximum contraction) of the inhibitory actions of DHA (3 × 10^−5^ M) on GFSM contractions induced by CCh (6 × 10^−8^ M; A), Ang II (10^−7^ M; B), and BK (10^−6^ M; C). DHA significantly inhibited the maximum GFSM contractions induced by CCh and Ang II (Fig. 1Ac, Bc), but did not cause significant inhibition of the AUC (Fig. 1Ab, Bb). DHA significantly suppressed both the maximum BK-induced contractions (Fig. 1Cc) and the AUC (Fig. 1Cb). Furthermore, the inhibitory actions of DHA (10^−5^–10^−4^ M) on the BK-induced contractions were mostly concentration-dependent (Supplementary Fig. 1).

### Inhibitory effects of extracellular Ca^2+^ removal and VDCC inhibitor on GFSM contractions induced by CCh, Ang II, and BK

**Figure 2:**
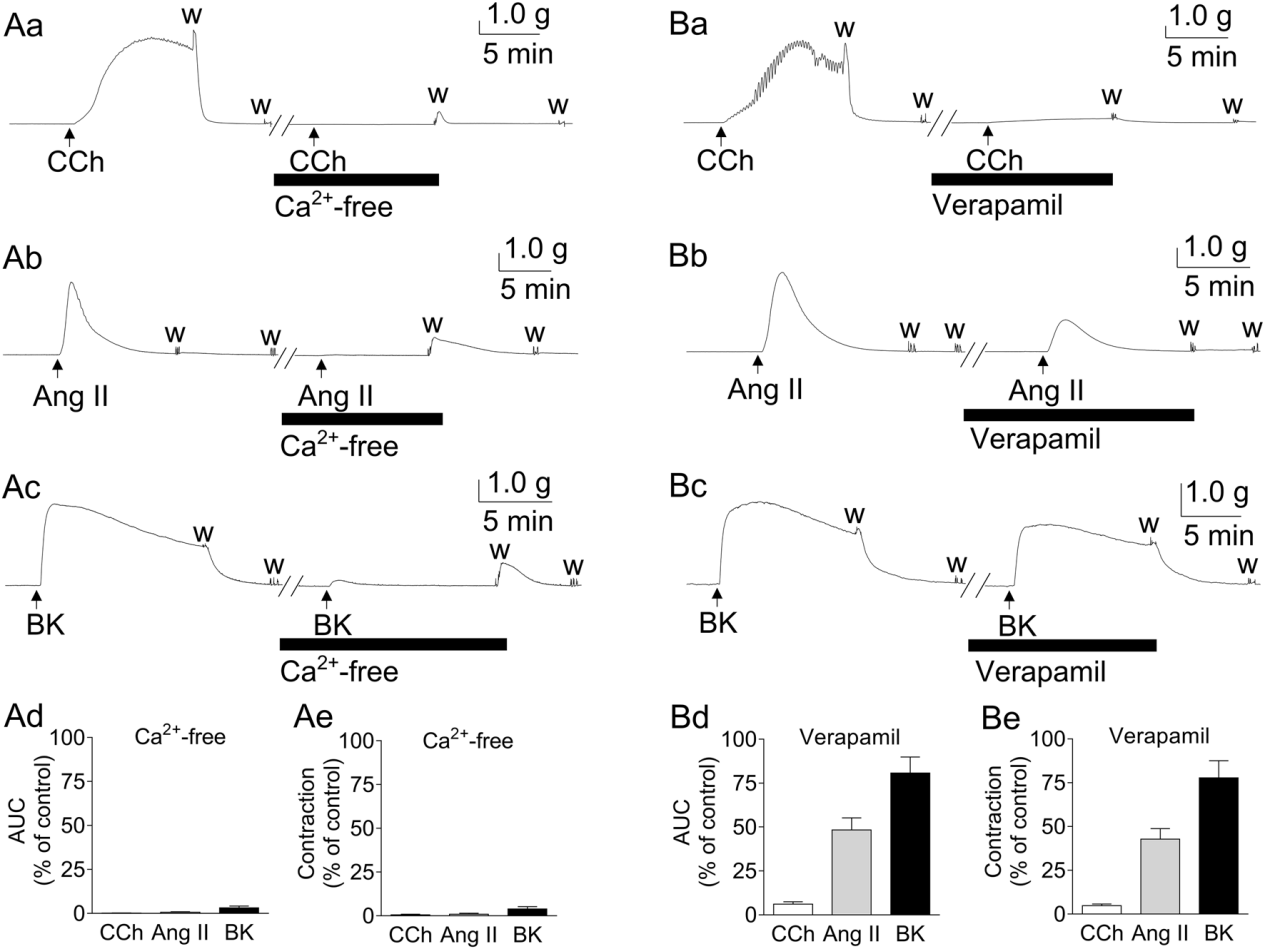
Representative traces (**a**–**c**) and summarized data (**d**: area under the curve (AUC); **e**: maximum contraction) of the inhibitory effects of extracellular Ca^2+^ removal (**A**) and verapamil (10^−5^ M; **B**) on the contractions induced by carbachol (CCh, 6 × 10^−8^ M; **a**), angiotensin II (Ang II, 10^−7^ M; **b**), and bradykinin (BK, 10^−6^ M; **c**) in guinea pig gastric fundus smooth muscle. Ca^2+^-free solution contained ethylene glycol-bis(2-aminoethylether)-*N*,*N*,*N′*,*N′*-tetraacetic acid (EGTA, 2 × 10^−4^ M). Data are expressed as means ± standard error of the mean (*n* = 5 (**A**, CCh in **B**), *n* = 10 (Ang II in **B**), *n* = 8 (BK in **B**)). w, wash out.

Figure 2A depicts the representative traces (a–c) and summarized data (d: AUC; e: maximum contraction) of the inhibitory actions of extracellular Ca^2+^ removal on GFSM contractions induced by CCh (6 × 10^−8^ M; a), Ang II (10^−7^ M; b), and BK (10^−6^ M; c). The contractions induced by CCh, Ang II, and BK were almost completely suppressed by replacing the extracellular fluid with Ca^2+^-free solution containing ethylene glycol-bis(2-aminoethylether)-*N*, *N*, *N′*, *N′*-tetraacetic acid (EGTA, 2 × 10^−4^ M).

Figure 2B depicts the representative traces (a–c) and summarized data (d: AUC; e: maximum contraction) of the inhibitory effects of a VDCC inhibitor (10^−5^ M verapamil) on GFSM contractions induced by CCh (6 × 10^−8^ M; a), Ang II (10^−7^ M; b), and BK (10^−6^ M; c). Unlike the effects of extracellular Ca^2+^ removal on GFSM contractions, the effects of verapamil varied greatly depending on the type of stimulant. CCh-induced contractions were potently inhibited by verapamil (inhibition of AUC: 93.9 ± 1.4%, *n* = 5), Ang II-induced contractions were inhibited to approximately 50% (inhibition of AUC: 51.6 ± 6.8%, *n* = 10), and BK-induced contractions were only partially inhibited (inhibition of AUC: 19.2 ± 9.1%, *n* = 8).

### Effects of receptor-operated Ca^2+^ channel (ROCC) and SOCC inhibitors on BK-induced GFSM contractions in the presence of VDCC inhibitor

**Figure 3:**
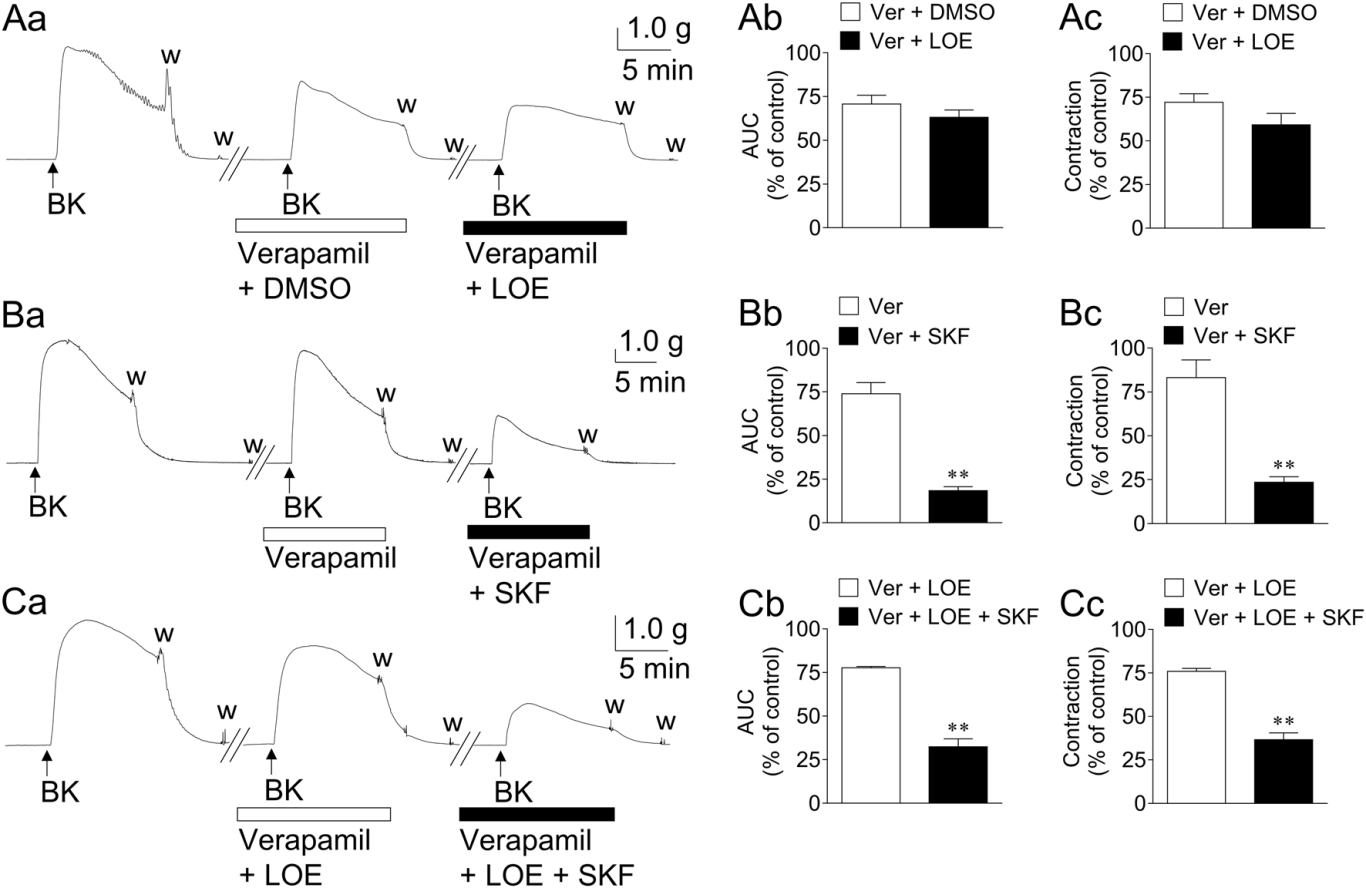
Representative traces (**a**) and summarized data (**b**: area under the curve (AUC); **c**: maximum contraction) of the effects of LOE-908 (LOE, 3 × 10^−5^ M; **A**) and SKF-96365 (SKF, 3 × 10^−5^ M; **B**, **C**) on the contractions induced by bradykinin (BK, 10^−6^ M) in the presence of verapamil (Ver, 10^−5^ M; **A**, **B**) or Ver plus LOE (**C**) in guinea pig gastric fundus smooth muscle. Data are expressed as means ± standard error of the mean (*n* = 9 (**A**, **B**), *n* = 5 (**C**)). **P < 0.01 versus Ver/Ver plus LOE (paired *t*-test). DMSO, dimethyl sulfoxide (0.015%), w, wash out.

Figure 3A and B depict the representative traces (a) and summarized data (b: AUC; c: maximum contraction) of the effects of an ROCC inhibitor (LOE-908, 3 × 10^−5^ M; A) and an ROCC/SOCC inhibitor (SKF-96365, 3 × 10^−5^ M; B) on GFSM contractions induced by BK (10^−6^ M) in the presence of the VDCC inhibitor verapamil (10^−5^ M). BK-induced contractions in the presence of verapamil were not significantly inhibited by LOE-908 (Fig. 3A) but were significantly inhibited by SKF-96365 (Fig. 3B) (inhibition from 74.0 ± 6.4 to 18.5 ± 2.2%, as assessed by AUC, *n* = 9).

Figure 3C depicts representative traces (a) and summarized data (b: AUC; c: maximum contraction) of the effects of SKF-96365 (3 × 10^−5^ M) on GFSM contractions induced by BK (10^−6^ M) in the presence of the combination of VDCC (10^−5^ M verapamil) and ROCC (3 × 10^−5^ M LOE-908) inhibitors. BK-induced contractions in the presence of both inhibitors were also significantly inhibited by SKF-96365 (Fig. 3C) (inhibition from 77.7 ± 0.7 to 32.4 ± 4.6%, as assessed by AUC, *n* = 5).

### Inhibitory actions of DHA on BK-induced GFSM contractions in the presence of VDCC and ROCC inhibitors

**Figure 4:**
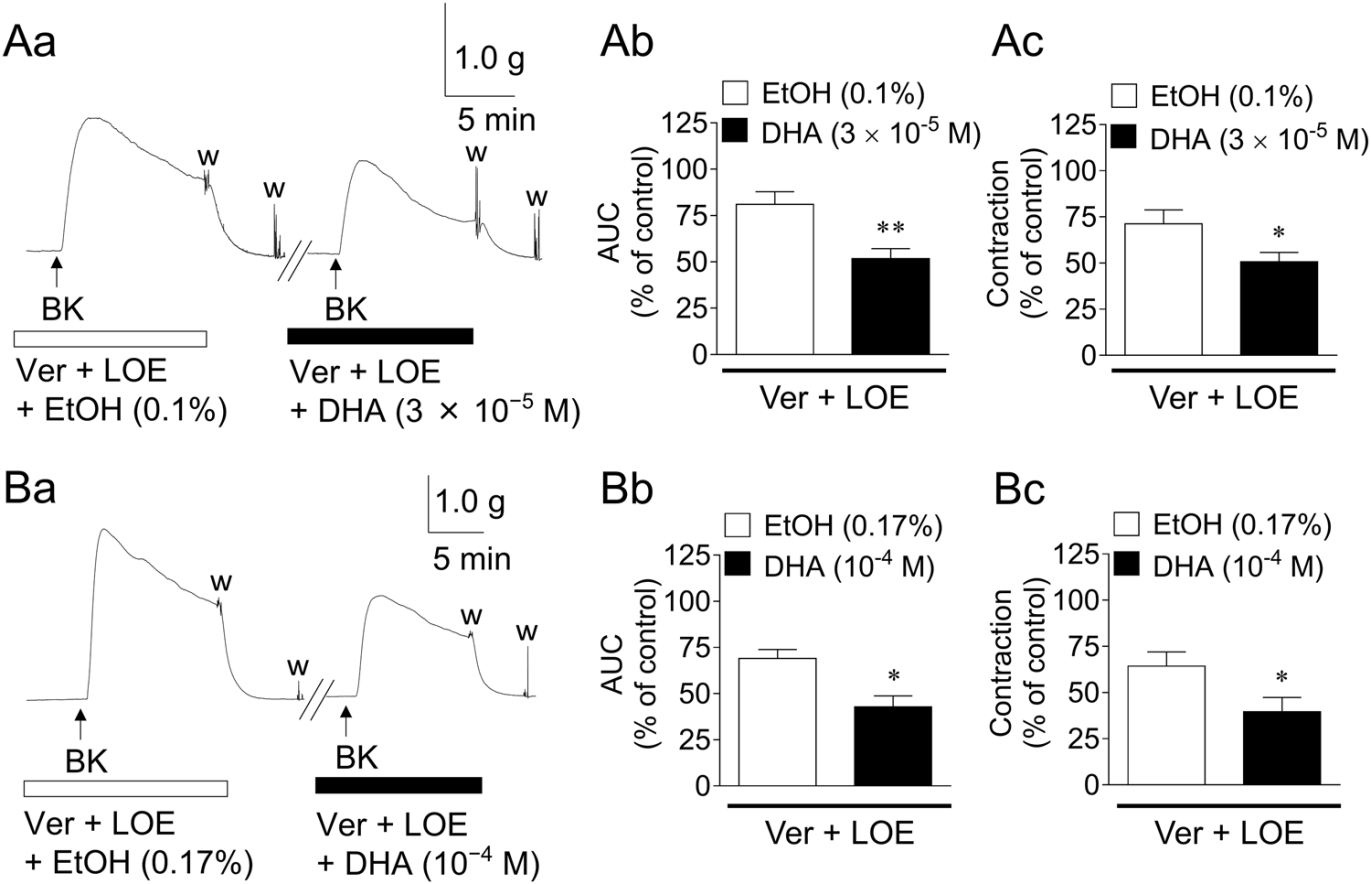
Representative traces (**a**) and summarized data (**b**: area under the curve (AUC); **c**: maximum contraction) of the inhibitory actions of docosahexaenoic acid (DHA, 3 × 10^−5^ M; **A** and DHA, 10^−4^ M; **B**) on the contractions induced by bradykinin (BK, 10^−6^ M) in the presence of verapamil (Ver, 10^−5^ M) plus LOE-908 (LOE, 3 × 10^−5^ M) in guinea pig gastric fundus smooth muscle. Data are expressed as means ± standard error of the mean (*n* = 6 each). *P < 0.05, **P < 0.01 versus EtOH (paired *t*-test). EtOH, ethanol; w, wash out.

Figure 4 depicts the representative traces (a) and summarized data (b: AUC; c: maximum contraction) of the inhibitory actions of DHA (3 × 10^−5^ M; A and 10^−4^ M; B) on GFSM contractions induced by BK (10^−6^ M) in the combined presence of a VDCC inhibitor (verapamil, 10^−5^ M) and ROCC inhibitor (LOE-908, 3 × 10^−5^ M). BK-induced contractions in the presence of VDCC and ROCC inhibitors were significantly suppressed by 35–40% by DHA (3 × 10^−5^ M and 10^−4^ M).

### Inhibitory actions of DHA on GFSM contractions induced by cyclopiazonic acid (CPA) in the presence of VDCC and ROCC inhibitors

**Figure 5:**
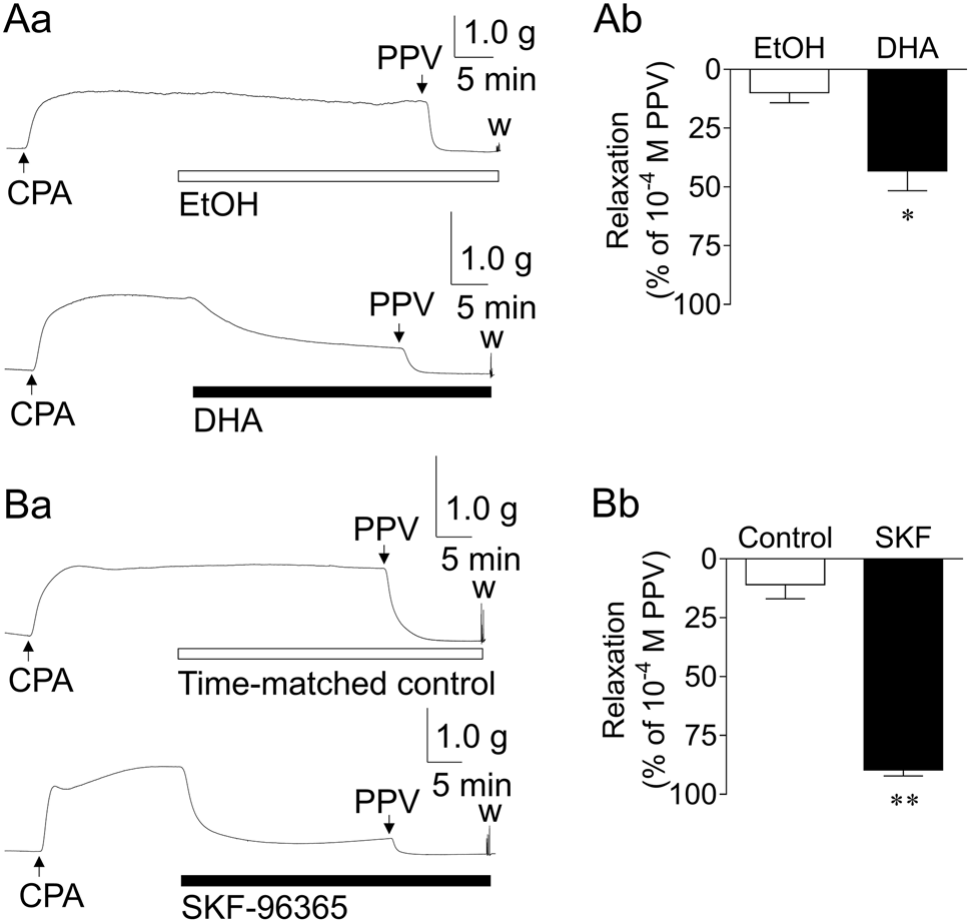
Representative traces (**a**) and summarized data (**b**) of the inhibitory actions of docosahexaenoic acid (DHA, 3 × 10^−5^ M; **A**) and SKF-96365 (SKF, 3 × 10^−5^ M; **B**) on the contractions induced by cyclopiazonic acid (CPA, 3 × 10^−5^ M) in the presence of verapamil (10^−5^ M) plus LOE-908 (3 × 10^−5^ M) in guinea pig gastric fundus smooth muscle. Data are expressed as means ± standard error of the mean (*n* = 6 (**A**), *n* = 5 (**B**)). *P < 0.05, **P < 0.01 versus EtOH/control (paired *t*-test). EtOH, ethanol (0.1%); PPV, papaverine (10^−4^ M); w, wash out.

Figure 5 depicts the representative traces (a) and summarized data (b) of the inhibitory actions of DHA (3 × 10^−5^ M; A) and SKF-96365 (3 × 10^−5^ M; B) on GFSM contractions induced by CPA (a sarco/endoplasmic reticulum (SER) Ca^2+^-ATPase (SERCA) inhibitor, 3 × 10^−5^ M) in the combined presence of a VDCC inhibitor (verapamil, 10^−5^ M) and ROCC inhibitor (LOE-908, 3 × 10^−5^ M). DHA and SKF-96365 significantly inhibited CPA-induced contractions; the relaxation rate (percent relaxation versus 10^−4^ M papaverine (PPV)-induced relaxation) was 43.2 ± 8.4% for DHA (*n* = 6) and 89.7 ± 2.5% for SKF-96365 (*n* = 5).

### Inhibitory actions of DHA on intracellular Ca^2+^ increase due to Ca^2+^ addition in CPA-treated 293T cells in Ca^2+^-free medium

**Figure 6:**
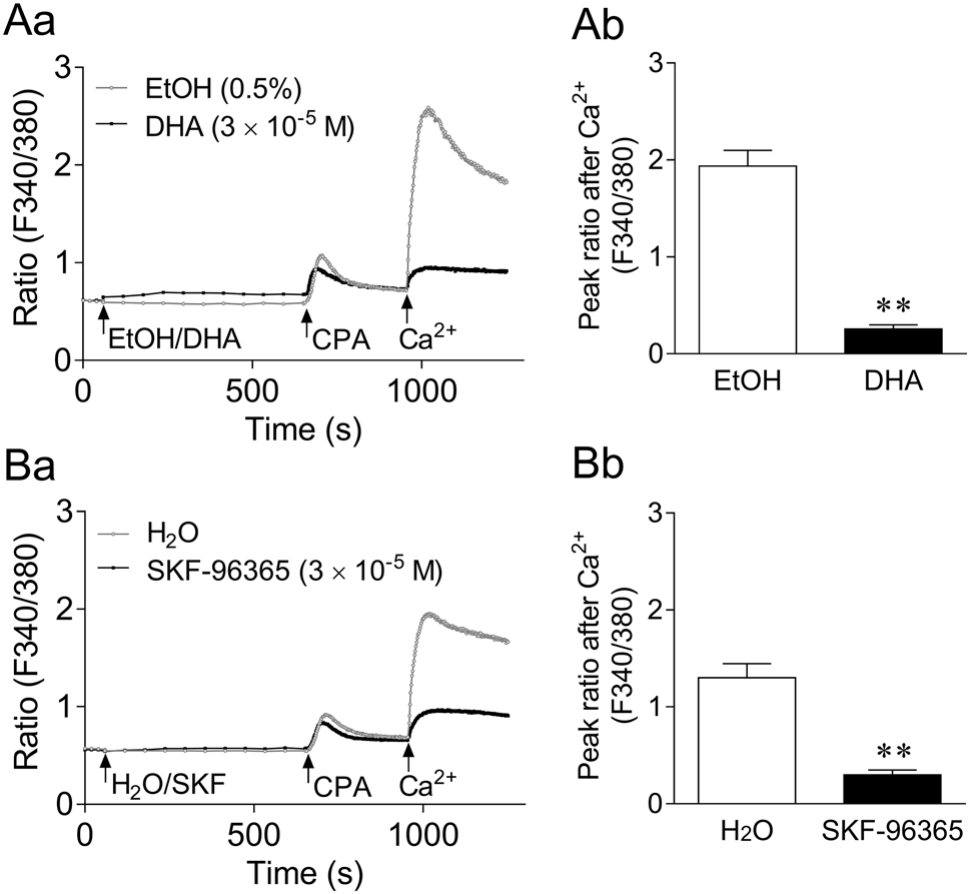
Inhibitory actions of docosahexaenoic acid (DHA, 3 × 10^−5^ M; **A**) and SKF-96365 (SKF, 3 × 10^−5^ M; **B**) on intracellular Ca^2+^ increase due to Ca^2+^ (1.8 mM) addition in cyclopiazonic acid (CPA, 10^−5^ M)-treated 293T cells in Ca^2+^-free medium. **a**: Mean Fura-2 fluorescence intensity ratio (F340/380) changes in the absence and presence of DHA (**Aa**) and SKF (**Ba**). Arrows indicate the administration of each drug. **b**: Summarized data of the peak ratio (F340/380) within 5 min after Ca^2+^ addition in the absence and presence of DHA (**Ab**) and SKF (**Bb**). Data are expressed as means ± standard error of the mean (*n* = 10 (**A**), n = 6 (**B**)). **P < 0.01 versus EtOH/H_2_O (Welch’s *t*-test). EtOH, ethanol.

Figure 6Aa and Ba depict the inhibitory actions of DHA (3 × 10^−5^ M; Aa) and SKF-96365 (3 × 10^−5^ M; Ba) on the intracellular Ca^2+^ increase due to Ca^2+^ (1.8 mM) addition in CPA (10^−5^ M)-treated 293T cells in Ca^2+^-free medium. Figure 6Ab and Bb show summarized data of the peak ratio (F340/380) within 5 min after Ca^2+^ addition in the absence and presence of DHA (Ab) and SKF-96365 (Bb). DHA and SKF-96365 significantly inhibited the intracellular Ca^2+^ increase due to Ca^2+^ addition; the inhibition rate was 86.7% for DHA and 77.0% for SKF-96365.

### Effects of BK receptor antagonists on BK-induced GFSM contractions and effects of DHA on BK-induced intracellular Ca^2+^ increase in BK B_2_ receptor-expressing 293T cells (B_2_-293T cells)

**Figure 7:**
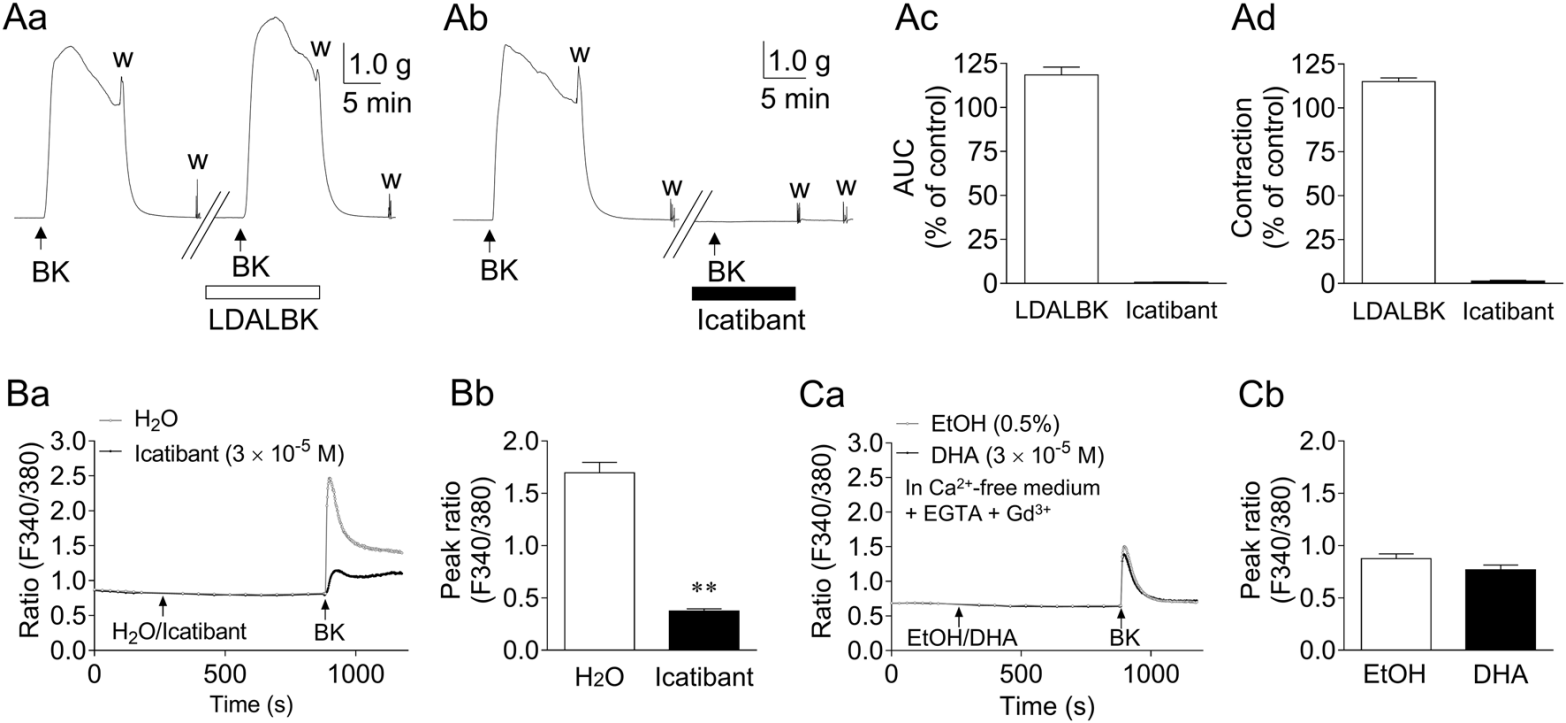
Effects of bradykinin (BK) receptor antagonists on BK-induced contractions in guinea pig gastric fundus smooth muscle (GFSM) (**A**), and effects of icatibant (**B**) and docosahexaenoic acid (DHA; **C**) on intracellular Ca^2+^ increase in BK B_2_ receptor-expressing 293T cells (B_2_-293T cells). **A**: Representative traces (**Aa**, **Ab**) and summarized data (**Ac**: area under the curve (AUC); **Ad**: maximum contraction) of the effects of Lys-(Des-Arg^9^, Leu^8^)-BK (LDALBK, 3 × 10^−5^ M; **Aa**) and icatibant (3 × 10^−5^ M; **Ab**) on BK (10^−6^ M)-induced GFSM contractions. **B**: Changes in mean Fura-2 fluorescence intensity ratio (F340/380) induced by BK (10^−6^ M) in the presence and absence of icatibant (3 × 10^−5^ M) in B_2_-293T cells in normal medium (**Ba**), and the summarized data of the peak ratio (F340/380) within 5 min after BK administration (**Bb**). **C**: Changes in mean Fura-2 fluorescence intensity ratio (F340/380) induced by BK (10^−6^ M) in the absence and presence of DHA (3 × 10^−5^ M) in B_2_-293T cells in Ca^2+^-free medium containing ethylene glycol-bis(2-aminoethylether)-*N*,*N*,*N′*,*N′*-tetraacetic acid (EGTA, 2 × 10^−4^ M) and Gd^3+^ (10^−5^ M) (**Ca**), and summarized data of the peak ratio (F340/380) within 5 min after BK administration (**Cb**). Arrows indicate the administration of each drug. Data are expressed as means ± standard error of the mean (*n* = 12 each). **P < 0.01 versus H_2_O (Welch’s *t*-test). EtOH, ethanol; w, wash out.

Figure 7A depicts the representative traces (Aa, Ab) and summarized data (Ac: AUC; Ad: maximum contraction) of the effects of BK receptor antagonists on GFSM contractions induced by BK (10^−6^ M). The BK receptor antagonists used in this study were Lys-(Des-Arg^9^, Leu^8^)-BK (LDALBK) (a BK B_1_ receptor antagonist, 3 × 10^−5^ M; Aa) and icatibant (a BK B_2_ receptor antagonist, 3 × 10^−5^ M; Ab). BK-induced contractions were not suppressed by LDALBK but were almost completely suppressed by icatibant.

Figure 7Ba and Ca depict the effects of icatibant (3 × 10^−5^ M; Ba) and DHA (3 × 10^−5^ M; Ca) on the intracellular Ca^2+^ increase induced by BK (10^−6^ M) in B_2_-293T cells. Figure 7Bb and Cb show summarized data of the peak ratio (F340/380) within 5 min after BK administration in the absence and presence of icatibant (Bb) and DHA (Cb). The experiments shown in Fig. 7C were performed in Ca^2+^-free solution containing EGTA (2 × 10^−4^ M) and Gd^3+^ (an ROCC inhibitor, 10^−5^ M) to eliminate extracellular Ca^2+^ influx. The BK-induced intracellular Ca^2+^ increase in B_2_-293T cells was significantly inhibited by icatibant (Fig. 7B). In contrast, DHA did not suppress the BK-induced intracellular Ca^2+^ increase in Ca^2+^-free solution containing EGTA and Gd^3+^ (Fig. 7C). However, in Ca^2+^-containing solution, DHA (3 × 10^−5^ M) suppressed the BK-induced intracellular Ca^2+^ increase (Supplementary Fig. 5).

### Measurement of SOCC-related mRNA expression levels

**Figure 8:**
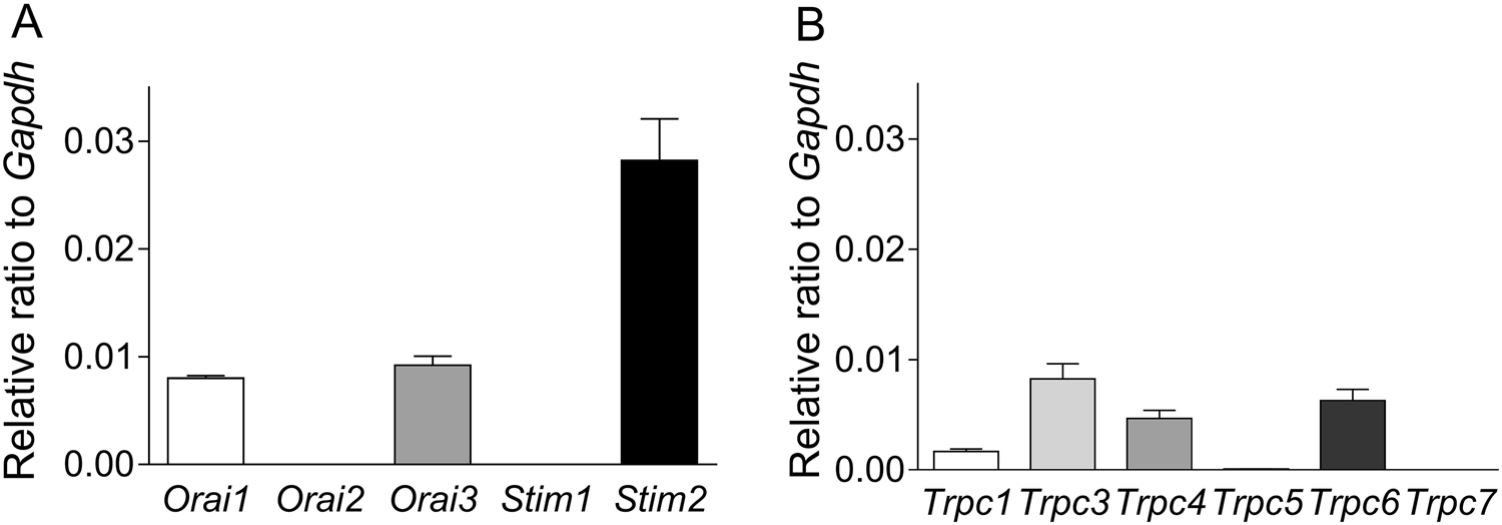
Expression levels of *Orai* (*Orai1*, *Orai2,* and *Orai3*) and *Stim* (*Stim1* and *Stim2*) mRNA (**A**) and *Trpc* (*Trpc1, 3–7*) mRNA (**B**) in guinea pig gastric fundus smooth muscle assessed by RT-qPCR. The expression level of each mRNA is shown relative to that of *Gapdh*, which is set as 1. Data are expressed as means ± standard error of the mean (*n* = 5 each).

Figure 8 depicts the relative SOCC-related mRNA expression levels in GFSM. We measured *Orai* (*Orai1–3*) mRNA, *Stim* (*Stim1* and *Stim2*) mRNA (Fig. 8A), and *Trpc* (*Trpc1, 3–7*) mRNA (Fig. 8B) as SOCC-related molecules. Among the *Orai* mRNA homologs, *Orai1* and *Orai3* were expressed to the same extent, and *Orai2* clearly had the lowest expression. Between the *Stim* mRNA homologs, *Stim2* was abundantly expressed, and *Stim1* clearly had the lowest expression. Among the *Trpc* mRNA homologs, *Trpc3* was abundantly expressed, followed by *Trpc6*, *Trpc4*, and *Trpc1*, while *Trpc5* and *Trpc7* clearly had the lowest expression.

### Inhibitory actions of Orai/TRPC channel inhibitors on GFSM contractions induced by CPA in the presence of VDCC and ROCC inhibitors

Supplementary Fig. 6 depicts the representative traces (A) and summarized data (B) of the inhibitory actions of 2-aminoethoxydiphenyl borate (2-APB, a non-selective Orai/TRPC channel inhibitor, 5 × 10^−5^ M; Ab)^25^, Synta66 (a selective Orai1 inhibitor, 3 × 10^−5^ M; Ac)^26^, Pyr10 (a selective TRPC3 channel inhibitor, 3 × 10^−6^ M; Ad)^27^, ML204 (a selective TRPC4 channel inhibitor, 10^−5^ M; Ae)^28^, SAR7334 (a selective TRPC6 channel inhibitor, 10^−7^ M; Af)^29^, and their vehicle (0.15% DMSO; Aa) on GFSM contractions induced by CPA (3 × 10^−5^ M) in the combined presence of a VDCC inhibitor (verapamil, 10^−5^ M) and ROCC inhibitor (LOE-908, 3 × 10^−5^ M). CPA-induced contractions were significantly suppressed by 2-APB and almost completely suppressed by Synta66. In contrast, CPA-induced contractions were not significantly suppressed by Pyr10, ML204, and SAR7334.

## Discussion

We have proposed TP receptor antagonism and functional inhibition of VDCCs as the mechanisms by which DHA produces immediate inhibitory activity against SM contractions induced by a TP receptor agonist/prostanoids and non-prostanoid agonists^8–11,17,18^. In addition, we now show that inhibition of SOCC-mediated Ca^2+^ influx is a new mechanism responsible for the DHA-induced inhibition of SM contractions (Supplementary Fig. 7).

We previously reported that DHA suppressed GP GFSM contractions induced by U46619 (a TXA_2_ mimetic) and prostanoids^17^. In the present study, we showed that DHA significantly suppressed the maximum contractions induced by CCh/Ang II/BK, although this unsaturated fatty acid showed a significant inhibition only against BK when contractions were assessed by AUC (Fig. 1). Contractions induced by CCh/Ang II/BK were shown to be mediated through their corresponding receptor subtypes (acetylcholine M_3_, Ang II AT_1_, and BK B_2_, respectively) since they were almost abolished by their corresponding selective receptor antagonists (solifenacin^30^, losartan^31^, and icatibant^32^, respectively) (Supplementary Fig. 2, Fig. 7AB). In contrast, DHA did not inhibit intracellular Ca^2+^ increases in Ca^2+^-free solution containing EGTA and Gd^3+^ produced by these chemical receptor stimulants in cells expressing acetylcholine M_3_, Ang II AT_1_, or BK B_2_ receptors, respectively (Supplementary Fig. 3, Fig. 7C). In Ca^2+^-free solution, these chemical receptor stimulants increased intracellular Ca^2+^ concentrations due to Ca^2+^ release from intracellular Ca^2+^ stores, but not due to extracellular Ca^2+^ influx. Therefore, potential inhibition of these chemical receptors and Ca^2+^ release from intracellular Ca^2+^ stores were judged to be excluded as mechanisms by which DHA inhibits non-prostanoid stimulation-induced contractions of GFSM.

Other than chemical receptors, DHA’s targets for its inhibitory actions against non-prostanoid-induced contractions would include pathways of Ca^2+^ influx from extracellular spaces^18^. Indeed, we previously showed that functional suppression of VDCCs could partly account for the inhibitory actions of DHA versus acetylcholine-/histamine-induced contractions in ileal and colonic longitudinal SMs^18^. Therefore, we next examined the extracellular Ca^2+^ dependency and extent of inhibition by verapamil (a VDCC inhibitor)^33^ of GFSM contractions induced by CCh/Ang II/BK. All contractions induced by these chemicals were almost completely abolished by extracellular Ca^2+^ removal, indicating that these contractions almost completely depend on extracellular Ca^2+^ influx and not Ca^2+^ release from intracellular Ca^2+^ stores (Fig. 2A). However, the extent of inhibition by verapamil differed among the contractions (Fig. 2B); CCh-induced contractions were almost completely suppressed by verapamil, whereas those induced by Ang II and BK were suppressed by 52% and 19%, respectively. These results indicate that the extent of functional contribution of VDCCs is 100%, ~ 50%, and ~ 20% for GP GFSM contractions induced by CCh, Ang II, and BK, respectively. In addition to these findings, we previously showed that DHA significantly but partly inhibits high KCl-induced contractions of GP GFSM^17^. Therefore, functional inhibition of VDCCs may be partly but substantially responsible for the inhibitory actions of DHA on these non-prostanoid-induced contractions. This interpretation supporting the involvement of functional VDCC inhibition is consistent with our previously proposed mechanism by which DHA inhibited contractions induced by U46619 and various prostanoids in GP GFSM^17^.

In contrast to CCh-induced contractions, more than 50% of Ang II- and BK-induced contractions remained in the presence of verapamil (Ang II: 48%, BK: 81%). This finding suggests that Ca^2+^ influxes through non-VDCC pathways play a sub-principal (Ang II) or principal (BK) role in inducing GFSM contractions and that such Ca^2+^ influx pathways are inhibited by DHA. Especially, the finding that ~ 80% of BK-induced contraction remained in the presence of verapamil implies that suppression of BK-induced contractions by DHA was mediated by inhibition of Ca^2+^ influxes through non-VDCC pathways. Potential candidates for the non-VDCC Ca^2+^ influx pathways responsible for SM contractions are ROCCs and SOCCs^34^. Therefore, we next examined whether these Ca^2+^ channels are responsible for BK-induced GFSM contraction using their corresponding inhibitors. The results clearly showed that BK-induced contractions in the presence of verapamil were not inhibited by LOE-908 (an ROCC inhibitor)^35^ (Fig. 3A) but were strongly inhibited by SKF-96365 (an ROCC/SOCC inhibitor)^36^ (Fig. 3B, C ). Therefore, SOCCs may play an important role as the primary extracellular Ca^2+^ influx pathway to generate BK-induced contractions in GP GFSM. Furthermore, the finding that DHA (3 × 10^−5^ M, 10^−4^ M) significantly suppressed BK-induced contractions in the presence of verapamil plus LOE-908 (Fig. 4) suggests that SOCCs are a target of DHA to produce its inhibitory action against BK-induced contractions.

However, BK-induced contractions were not completely suppressed even in the presence of verapamil/LOE-908/SKF-96365 (Fig. 3C). The remaining contractile component may be dependent on extracellular Ca^2+^ influx through SOCCs, which could not be completely suppressed by SKF-96365 (3 × 10^−5^ M). Candidates other than SOCCs are LOE-908/SKF-96365-insensitive ROCCs and the reverse mode of the Na^+^/Ca^2+^ exchanger^37,38^.

In contrast, Ang II-induced contractions in the presence of verapamil were significantly inhibited by both LOE-908 and SKF-96365 (Supplementary Fig. 8). Thus, unlike BK-induced contractions, Ang II-induced contractions may be elicited by extracellular Ca^2+^ influx through ROCCs in addition to VDCCs/SOCCs.

To obtain more direct evidence to support the hypothesis that DHA exerts an immediate inhibitory effect on Ca^2+^ influx through SOCCs, we subsequently conducted two types of experiments with GP GFSM tissues and cultured 293T cells and obtained the following results. (1) SOCCs are the extracellular Ca^2+^ influx pathways, whose activation is triggered by Ca^2+^ depletion in the SER^39^. Therefore, chemical depletors such as SERCA inhibitors, including CPA, increase intracellular Ca^2+^ levels following activation of extracellular Ca^2+^ influx through SOCCs^40,41^, thus generating SM contractions^42^. In GP GFSM tissues, CPA induced contractions in the presence of verapamil and LOE-908 (VDCC and ROCC inhibitors, respectively). Since CPA-induced contractions were almost completely inhibited by SKF-96365, such contractions were shown to be caused by extracellular Ca^2+^ influx through SOCCs (Fig. 5B). The finding that the SKF-96365-inhibitable contractions induced by CPA were strongly attenuated by DHA indicated that DHA was able to inhibit the extracellular Ca^2+^ influx through SOCCs (Fig. 5A). (2) More direct evidence was obtained by Ca^2+^ measurements in 293T cells. In these cells, an SKF-96365-inhibitable increase in Ca^2+^ concentration was generated by Ca^2+^ addition following CPA treatment in Ca^2+^-free medium. This indicated that this intracellular Ca^2+^ increase was induced by Ca^2+^ influx through SOCCs, the activation of which was triggered by CPA-induced depletion of SER Ca^2+^ (Fig. 6B). DHA again strongly suppressed this intracellular Ca^2+^ increase, indicating that DHA could inhibit SOCC-mediated extracellular Ca^2+^ influx (Fig. 6A). Note that (i) this intracellular Ca^2+^ increase was unaffected by LOE-908^43^, and (ii) in a separate series of experiments, the CPA-induced intracellular Ca^2+^ increase in Ca^2+^-containing medium was unaffected by verapamil plus LOE-908 (Supplementary Fig. 4). These results exclude the potential contribution of VDCCs and ROCCs. To the best of our knowledge, this is the first report indicating the potential inhibitory action of DHA against SOCCs that are responsible for contraction of SM tissues. To confirm that DHA more directly inhibits drug receptor-stimulated Ca^2+^ entry through SOCCs, the effects of DHA on intracellular Ca^2+^ increases following depletion of Ca^2+^ stores after repeated stimulation of the drug receptor should be examined in the future to complement the use of CPA.

One type of SOCC is composed of Orai channels, which are the main Ca^2+^ entry channels, and stromal interaction molecules (STIMs), which are SER Ca^2+^ sensors that control Orai activation^34^. There are three isoforms of Orai channels (Orai1–3) and two isoforms of STIMs (STIM1, 2). In this study, we measured the mRNA expression of these isoforms in GFSM and found relatively high expression of *Orai1*, *Orai3*, and *Stim2* (Fig. 8A). Transient receptor potential canonical (TRPC) channels represent another type of SOCC^44^. We also measured the mRNA expression of TRPC channel isoforms (*Trpc1*, *Trpc3–7*) in GFSM and found relatively high expression in the following order: *Trpc3* > *Trpc6* > *Trpc4* > *Trpc1* (Fig. 8B). To estimate the functional SOCC molecules in GP GFSM, we investigated the effects of five types of Orai/TRPC channel inhibitors on the CPA-induced contractions. CPA-induced contractions in the presence of verapamil and LOE-908 were significantly suppressed by 2-APB (a non-selective Orai/TRPC channel inhibitor) and almost completely suppressed by Synta66 (a selective Orai1 inhibitor), but not Pyr10 (a selective TRPC3 channel inhibitor), ML204 (a selective TRPC4 channel inhibitor), and SAR7334 (a selective TRPC6 channel inhibitor) (Supplementary Fig. 6). Since LOE-908 has been reported to inhibit TRPC1^45^, the involvement of TRPC1 can be excluded under these conditions. The involvement of Orai3 could not be investigated in this study because a selective inhibitor is not available. However, since the CPA-induced contractions were almost completely abolished by the selective Orai1 inhibitor (Synta66), the contribution of Orai3 should be negligible. Therefore, in GP GFSM, the depletion of SER Ca^2+^ may activate STIM2, followed by the activation of Orai1. Although the detailed mechanisms by which DHA suppresses SOCCs are still unknown, two possibilities exist: (1) DHA directly suppresses Orai channels (Orai1) or (2) DHA suppresses the activation of Orai channels (Orai1) by STIM (STIM2). More detailed information can be obtained with SOCC-related gene knockout animals or knockdown GFSM cells.

In conclusion, to the best of our knowledge, we are the first to find that DHA can inhibit GP GFSM contractions mediated through non-prostanoid receptor agonists. The mechanisms by which DHA inhibits these contractions involve inhibition of Ca^2+^ influx through SOCCs, which may be largely responsible for the inhibition of BK-induced contractions.

## Methods

### Animals

We used male GPs (weight: 310–690 g, age: 4–16 weeks; Kyudo Co., Ltd., Saga, Japan), which were housed under a fixed 12/12 h light/dark cycle (08:00–20:00) and controlled conditions (20–22 °C, relative air humidity: 50 ± 5%) with food and water available ad libitum. This study was carried out in compliance with ARRIVE guidelines and the guidelines of the Laboratory Animal Center of Faculty of Pharmaceutical Sciences, Toho University. This study was approved by the Toho University Animal Care and User Committee (approval number: 20–444).

### GFSM strips

GFSM strips were prepared and tension changes were recorded according to our previous report^17^. GPs were anesthetized with isoflurane (inhalation) and exsanguinated from the carotid artery. The isolated stomach was denuded of its connective and adipose tissues, and then separated into the GF and gastric body in Locke–Ringer solution containing (in mM) NaCl, 154; KCl, 5.6; CaCl_2_, 2.2; MgCl_2_, 2.1; NaHCO_3_, 5.9; and d-(+)-glucose, 2.8. The GF interior was irrigated with the solution. The GF was further cut along the longitudinal axis into 2–4 segments, and the epithelium of the segments was removed gently. The GFSM strips were approximately 5–20 mm in length and 2–3 mm in width.

The GFSM strips were suspended under a 1.0-g resting tension with clips and cotton thread in a 20-ml organ bath filled with Locke–Ringer solution. The solution was maintained at 32 ± 1 °C and oxygenated with 95% O_2_ and 5% CO_2_. The suspended strips were allowed to equilibrate for 60 min. Tension changes of GFSM strips were isometrically recorded with PowerLab™ and LabChart™ (Version 7) software (ADInstruments Pty. Ltd., Bella Vista, NSW, Australia) using force–displacement transducers (TB-612T, Nihon Kohden, Tokyo, Japan; FORT 25, World Precision Instruments, Sarasota, FL, USA) and carrier amplifiers (MSC-2 Signal Conditioner, Labo Support Co., Osaka, Japan; AP-621G, Nihon Kohden; TBM4M, World Precision Instruments). After a 60-min incubation, the strips were contracted by CCh (10^−5^ M) at least three times with a 10-min interval. Thereafter, the strips were contracted by CCh (6 × 10^−8^ M), Ang II (10^−7^ M), and BK (10^−6^ M) for 10 min at least twice with an interval of 30 min (CCh/BK) or 120 min (Ang II) until stable contractions were obtained. To prevent the potential action of endogenous prostaglandins, all tension recordings were carried out in the presence of indomethacin (3 × 10^−6^ M).

### Effects of DHA and various Ca^2+^ channel inhibitors on GFSM contractions

After stable contractions by the tested drug were obtained, ethanol (EtOH: the DHA vehicle, 0.1%), verapamil (10^−5^ M), verapamil plus dimethyl sulfoxide (DMSO: the LOE-908 vehicle, 0.05%), verapamil plus LOE-908 (an ROCC inhibitor, 3 × 10^−5^ M), or verapamil plus LOE-908 plus EtOH (the DHA vehicle, 0.1%/0.17%) was applied to the bath solution. After a 30-min incubation, the strips were contracted by the tested drug for 10 min. After washing out, DHA (3 × 10^−5^ M), verapamil plus LOE-908, verapamil plus SKF-96365 (an SOCC/ROCC inhibitor, 3 × 10^−5^ M), verapamil plus LOE-908 plus SKF-96365, or verapamil plus LOE-908 plus DHA (3 × 10^−5^ M/10^−4^ M) was added to the bath solution. After a 30-min incubation, the strips were contracted using the tested drug for 10 min. For Ang II-induced contractions, EtOH/verapamil/DHA was added after a 90-min incubation.

### Effects of extracellular Ca^2+^ removal on GFSM contractions

After stable contractions were obtained, the strips were incubated for 20 min (CCh/BK) or 110 min (Ang II). After the 20- or 110-min incubation, the bath solution was replaced with Ca^2+^-free solution containing (in mM) NaCl, 154; KCl, 5.6; MgCl_2_, 2.1; NaHCO_3_, 5.9; d-(+)-glucose, 2.8; and EGTA, 0.2. After a 10-min incubation, the strips were contracted using the tested drug for 10 min.

### Effects of DHA and SKF-96365 on CPA-induced GFSM contractions

The GFSM strips were suspended according to the procedures described in “GFSM strips.” After a 60-min incubation, the bath solution was replaced with 80 mM KCl solution containing (in mM) NaCl, 79.6; KCl, 80; CaCl_2_, 2.2; MgCl_2_, 2.1; NaHCO_3_, 5.9; and d-(+)-glucose, 2.8 for 10 min three times with a 10-min interval. Thereafter, verapamil (10^−5^ M) plus LOE-908 (3 × 10^−5^ M) was added in the bath solution. After a 10-min incubation, the strips were contracted by CPA (3 × 10^−5^ M) for ≥ 20 min. When the contractions stabilized, DHA (3 × 10^−5^ M), SKF-96365 (3 × 10^−5^ M), or their vehicle (0.1% EtOH/H_2_O) was added in the bath solution. After a 30-min incubation, the strips were relaxed by PPV (10^−4^ M).

### Measurement of intracellular Ca^2+^ changes

The measurement of intracellular Ca^2+^ changes was performed as previously described^10^. Briefly, the day before the measurement, 293T and B_2_-293 T cells were seeded at ~ 90% confluence and cultured overnight. The next day, these cells were incubated in the presence of Fura-2 AM for 60 min. The cells were then rinsed with the medium. In the experiment shown in Fig. 6, the medium was replaced with Ca^2+^-free medium, and in the experiment shown in Fig. 7B, the medium was replaced with Ca^2+^-free medium containing EGTA (2 × 10^−4^ M)- and Gd^3+^ (10^−5^ M). After this procedure, the fluorescence intensity at 510 nm emission generated by 340 nm and 380 nm excitation was measured using microplate readers (Nivo, PerkinElmer Inc., Waltham, MA, USA; Infinite F200 Pro, Tecan Group Ltd., Mӓnnedorf, Switzerland). We assumed that the changes in the ratio of fluorescence intensities at 510 nm emission generated by 340 nm and 380 nm excitation (F340/380) reflected the relative changes in intracellular Ca^2+^ concentration. After a 10-min incubation in the presence of DHA (3 × 10^−5^ M), icatibant (3 × 10^−5^ M), or DHA/icatibant vehicle (0.5% EtOH/H_2_O), BK (10^−6^ M) or CPA (10^−5^ M) was added via the injector module, and the fluorescence intensity was measured for 5 min. After CPA application, Ca^2+^ (1.8 mM) was added via the injector module, and the fluorescence intensity was measured for 5 min.

At the end of the experiment, to determine background fluorescence, ionomycin (5 × 10^−6^ M) and Mn^2+^ (5 × 10^−2^ M) were added. This background fluorescence was subtracted from the fluorescence intensities of all measurements.

### RT-qPCR of SOCC-related mRNA expression

RT-qPCR was performed as previously described^17^. Briefly, total RNA was isolated from GFSM. First-strand cDNA was synthesized using ReverTra Ace® qPCR RT Master Mix with gDNA Remover (TOYOBO Co. Ltd., Osaka, Japan). RT-qPCR was performed on a CronoSTAR™ 96 Real-Time PCR System (Takara Bio Inc., Shiga, Japan) using Taq Pro Universal SYBR® qPCR Master Mix (NIPPON Genetics Co. Ltd., Tokyo, Japan). Supplementary Table 1 shows the primers used in this study. The thermal cycler parameters were set to 95 °C for 30 s, followed by 40 cycles of 95 °C for 10 s and 60 °C for 30 s. The fluorescence intensities were measured at each 60 °C step to confirm DNA amplification. We used CronoSTAR™ 96 Software (Takara Bio Inc.) to analyze the mRNA expression level of each gene. The mRNA expression levels were normalized to that of *glyceraldehyde-3-phosphate dehydrogenase* (*Gapdh*), which was set to 1. Samples that did not produce a Ct value after 40 cycles were considered to have no expression.

### Drugs

We used the following drugs in this study: CCh chloride, indomethacin, (±)-verapamil, and Synta66 (Sigma-Aldrich Co., St. Louis, MO, USA); DHA and solifenacin succinate (Tokyo Chemical Industry Co., Ltd., Tokyo, Japan); CPA, PD 123,319, LDALBK, ML204, and SAR7334 hydrochloride (Cayman Chemical Co., Ann Arbor, MI, USA); PPV, losartan, and 2-APB (FUJIFILM Wako Pure Chemical Co., Osaka, Japan); LOE-908 (Tocris Bioscience, Bristol, UK; Nippon Boehringer Ingelheim Co., Ltd., Hyogo, Japan; or synthesized in our facility); SKF-96365 (Tokyo Chemical Industry Co., Ltd.; Cayman Chemical Co.); icatibant acetate (Biosynth Ltd., Berkshire, UK); and Pyr10 (AdooQ Bioscience LLC., Irvine, CA, USA).

DHA/indomethacin was dissolved in EtOH to prepare a stock solution of 3 × 10^−2^ M/10^−2^ M. LOE-908/CPA/2-APB/Synta66/Pyr10/ML204/SAR7334 was dissolved in DMSO to prepare a stock solution of 6 × 10^−2^ M/1.5 × 10^−2^ M/1.67 × 10^−2^ M/2 × 10^−2^ M/2 × 10^−3^ M/6.67 × 10^−3^ M/6.67 × 10^−5^ M. All other drugs were dissolved in and diluted with distilled water.

We carried out preliminary studies and determined the concentration of each agonist (CCh, Ang II, and BK) that generated reproducible contractions of approximately 50% of the CCh (10^−5^ M)-induced contraction. The concentration of DHA was 3 × 10^−5^ M throughout this study (except that 10^−5^–10^−4^ M was used for Supplementary Fig. 1); our previous studies showed that this DHA concentration clearly exhibited TP receptor antagonistic action. The concentrations of verapamil^33^, LOE-908^35^, SKF-96365^46^, LDALBK^47^, icatibant^32^, solifenacin^30^, losartan^31^, and PD 123319^48^ were sufficient to inhibit their corresponding targets. These drugs had no obvious effects on the basal tension of GFSM.

### Data analysis

The contractions and AUC were analyzed with LabChart™. The contractions induced by each drug were analyzed at the maximum contraction for 10 min. AUC was analyzed for 10 min after the application of each drug. The contractions and AUC in the presence of the tested drugs were normalized to those in the absence of the tested drugs, which was set to 100%.

The extent of relaxation induced by DHA/SKF-96365/2-APB/Synta66/Pyr10/ML204/SAR7334 on CPA-induced contractions was calculated relative to the steady-state tension level prior to the application of DHA/SKF-96365/2-APB/Synta66/Pyr10/ML204/SAR7334 (0% relaxation) and to the tension level after the application of 10^−4^ M PPV (100% relaxation).

Data are expressed as means ± standard error of the mean (SEM), where *n* refers to the number of experiments. Statistical analyses were performed with paired *t*-tests, unpaired *t*-tests with Welch’s correction if necessary, or one-way ANOVA followed by Dunnett’s test, using GraphPad Prism™ (Version 6.0) (GraphPad Software, Inc., San Diego, CA, USA). Statistical significance was set at *P* < 0.05.

### Supplementary Information

Supplementary Information.

## Data Availability

The data that support the findings of this study are available from the corresponding author, K.O., upon reasonable request.
