# Supplementary material for: Inhibitory mechanisms of docosahexaenoic acid on carbachol-, angiotensin II-, and bradykinin-induced contractions in guinea pig gastric fundus smooth muscle
Source: Sci Rep. 2024 May 22;14:11720. doi: 10.1038/s41598-024-62578-y (PMC11111694; doi:10.1038/s41598-024-62578-y)
Supplement: Supplementary file 1 — Supplementary Information. [file 41598_2024_62578_MOESM1_ESM.pdf]

Supplementary Table and Figures

Scientific Reports / Article

Inhibitory mechanisms of docosahexaenoic acid on carbachol-, angiotensin II-, and bradykinin-induced contractions in guinea pig gastric fundus smooth muscle

Keyue Xu[1], Miyuki Shimizu[1], Toma Yamashita[1], Mako Fujiwara[1], Shunya Oikawa[1], Guanghan Ou[1], Naho Takazakura[1], Taichi Kusakabe[2], Keisuke Takahashi[2], Keisuke Kato[2], Kento Yoshioka[1], Keisuke Obara[1,*], Yoshio Tanaka[1]

[1]Department of Chemical Pharmacology, Faculty of Pharmaceutical Sciences, Toho University, Miyama 2-2-1, Funabashi-City, Chiba 274-8510, Japan
[2]Department of Organic Chemistry, Faculty of Pharmaceutical Sciences, Toho University, Miyama 2-2-1, Funabashi-City, Chiba 274-8510, Japan

***Correspondence to:**
Keisuke Obara, Ph.D.
Department of Chemical Pharmacology
Faculty of Pharmaceutical Sciences, Toho University
Miyama 2-2-1, Funabashi-City
Chiba 274-8510, Japan
E-mail: keisuke.obara@phar.toho-u.ac.jp

Supplementary Table 1 Primers used for RT-qPCR

IUPHAR† name	Gene symbol	Sequence (5′–3′)
Orai1	*Orai1*	GCATCAAGGTCTGGACACCG
		CTTCGCCGTGCATTTCTACC
Orai2	*Orai2*	GCTCAGGTACAGCTTCCTCC
		CTGCGCGGCAACGAATG
Orai3	*Orai3*	AGGCCAGCTCCACATAATGG
		GTCTCCACGTGTCTACTGCC
-	*Stim1*	AGCCTCGCCCTCTACCAAC
		TGTCAACAACCCTGGCATCC
-	*Stim2*	CTCGCTGAGACTGTGGTGAG
		TGGTGATGCCCAGAGTTTCC
TRPC1	*Trpc1*	ATGACGTGAGGAGAGAGCCG
		CGAGGTCGATGGGAGAGGAG
TRPC3	*Trpc3*	CGACGACTTCTACGCCTACG
		TGTGCACCACCTCGTACTTC
TRPC4	*Trpc4*	GATAACGAAGAAGAAGTTGCTCG
		CTGAGACCGGGAATGCTCAG
TRPC5	*Trpc5*	TTAGCGCTCAGGATTGCTCTG
		GGTGTCCCTCTTGCCTGTAG
TRPC6	*Trpc6*	CCCTAGCCAGTCTGAACTCC
		TCATGAGGCCGTTCAATCCG
TRPC7	*Trpc7*	ATGCTCTCCATCTTCGTGGC
		TCTGAAGGCCACCACTTGTC
-	*Gapdh*	ACGGATTTGGCCGTATTGGA
		CCATTCTCAGCCTTGACGGT

†International Union of Basic and Clinical Pharmacology. -: No IUPHAR name exists.

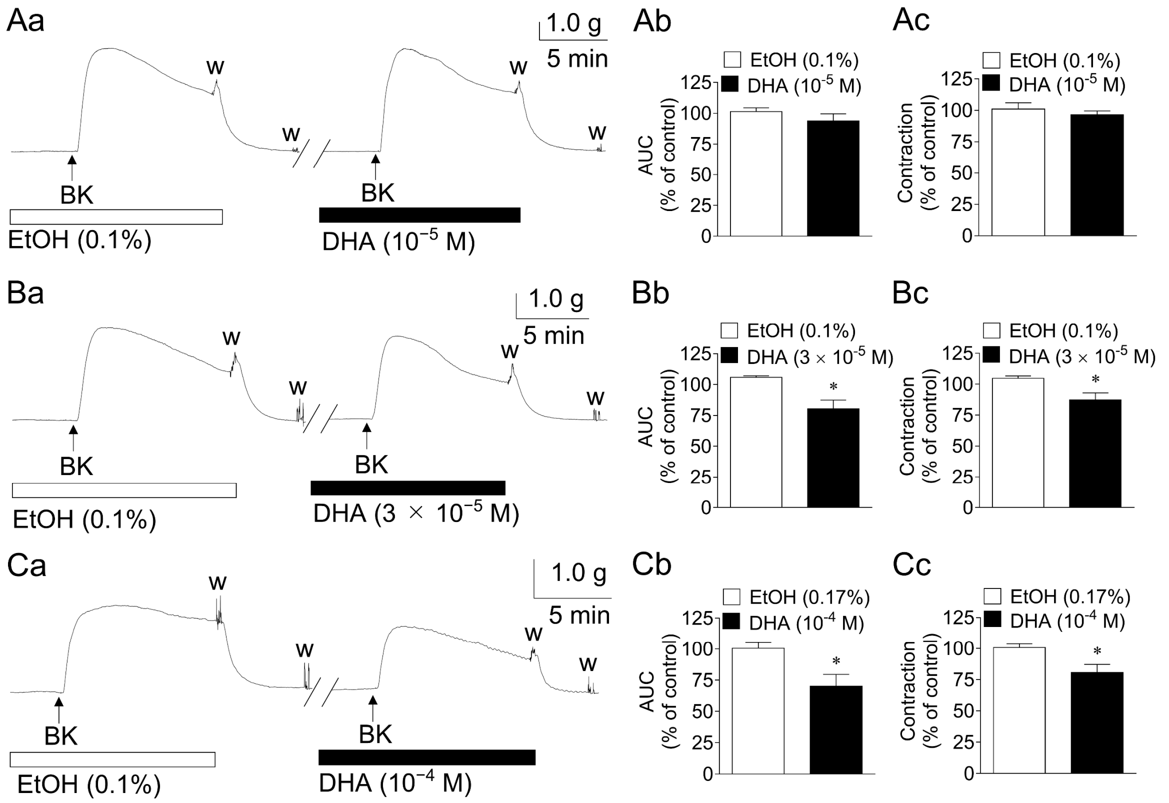

Supplementary Fig. 1

Representative traces (a) and summarized data (b: area under the curve (AUC); c: maximum contraction) of the inhibitory actions of docosahexaenoic acid (DHA, 10^{-5} M, 3×10^{-5} M, and 10^{-4} M; A, B, and C, respectively) on the contractions induced by bradykinin (BK, 10^{-6} M) in guinea pig gastric fundus smooth muscle. Data are expressed as means ± standard error of the mean (n = 5 (A, B), n = 6 (C)). *P < 0.05 *vs.* EtOH (paired Student's *t*-test). EtOH: ethanol, w: wash out.

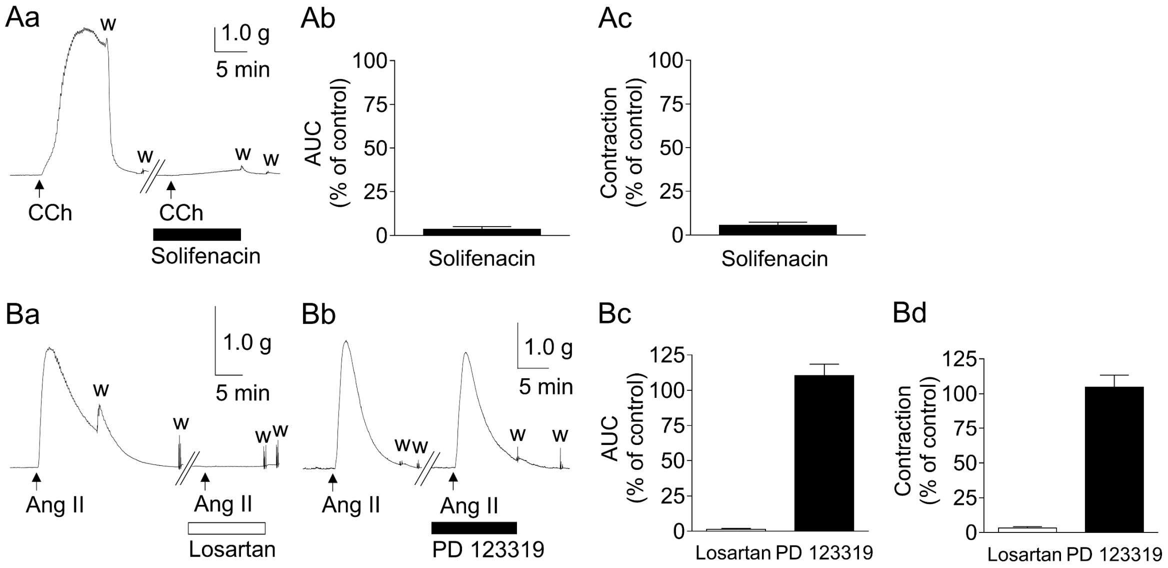

Supplementary Fig. 2

Representative traces (Aa, Ba, Bb) and summarized data (Ab, Bc: area under the curve (AUC); Ac, Bd: maximum contraction) of the inhibitory effects of solifenacin (3×10^{-8} M, an acetylcholine M_3 receptor antagonist; A), losartan (10^{-6} M, an angiotensin II (Ang II) AT_1 receptor antagonist; Ba), and PD 123319 (10^{-6} M, an Ang II AT_2 receptor antagonist; Bb) on the contractions induced by carbachol (CCh, 6×10^{-8} M; A) and Ang II (10^{-7} M; B) in guinea pig gastric fundus smooth muscle. Data are expressed as means ± standard error of the mean (n = 5 each). w: wash out.

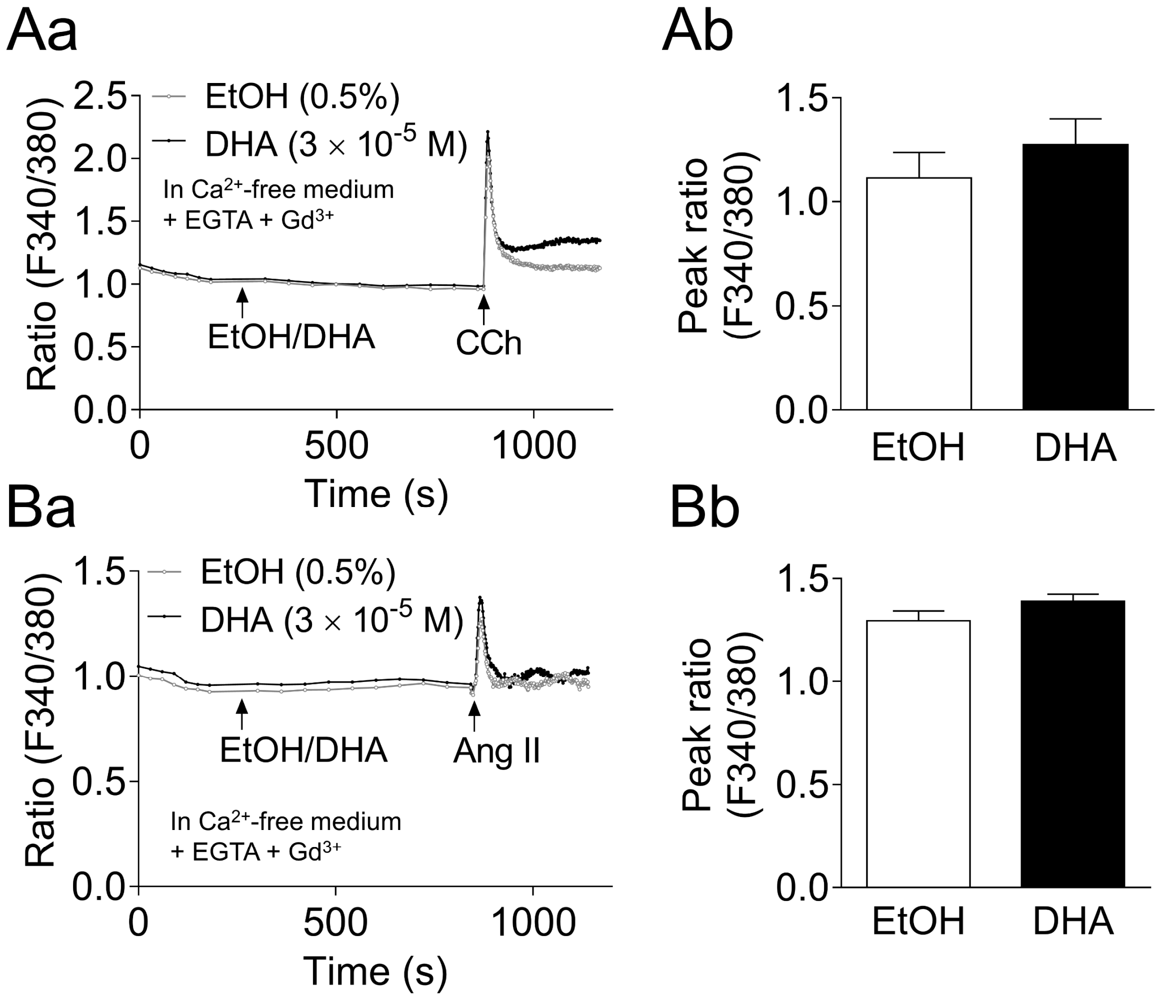

Supplementary Fig. 3

Effects of docosahexaenoic acid (DHA, 3×10^{-5} M) on carbachol (CCh, 10^{-6} M)-induced intracellular Ca^{2+} increase in acetylcholine M_3 receptor-expressing 293T cells (A) and angiotensin II (Ang II, 10^{-7} M)-induced intracellular Ca^{2+} increase in Ang II AT_1 receptor-expressing 293T cells (B). a: Changes in mean Fura-2 fluorescence intensity ratio (F340/380) in the presence and absence of DHA. Arrows indicate the administration of each drug. b: Summarized data of the peak ratio (F340/380) within 5 min after CCh (A) and Ang II (B) administration in the absence and presence of DHA. Data are expressed as means ± standard error of the mean (n = 10 (A), n = 3 (B)). EGTA: ethylene glycol-bis(2-aminoethylether)-*N,N,N′,N′*-tetraacetic acid, EtOH: ethanol.

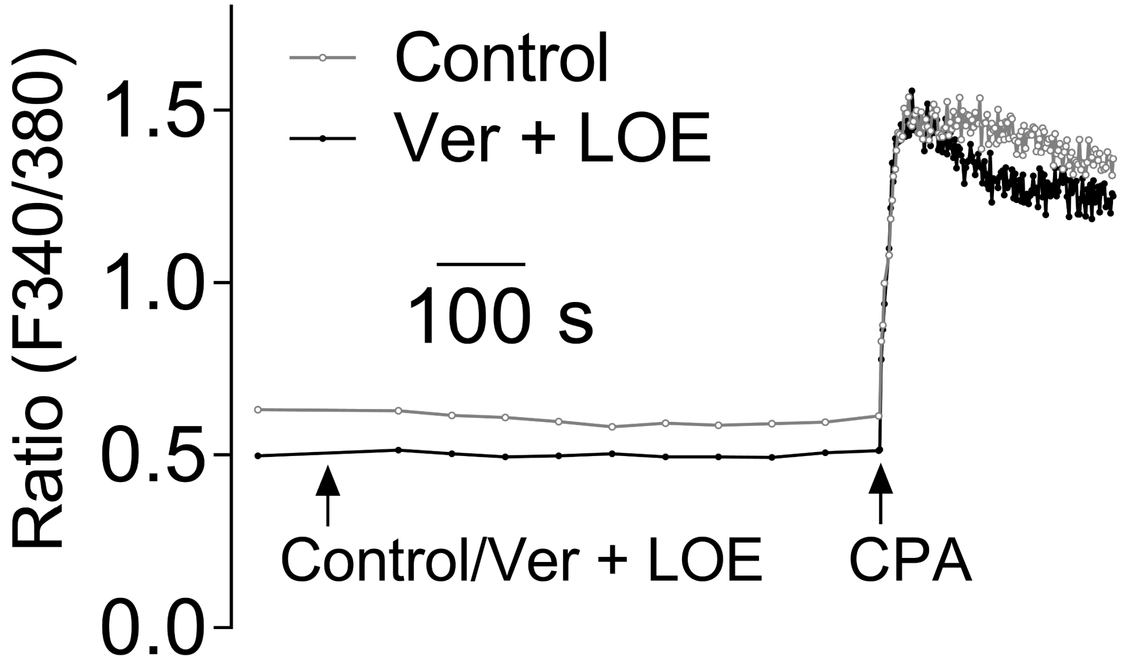

Supplementary Fig. 4

Effects of verapamil (Ver, 10^{-5} M) plus LOE-908 (LOE, 3 × 10^{-5} M) on intracellular Ca^{2+} increase induced by cyclopiazonic acid (CPA, 10^{-5} M) in 293T cells in Ca^{2+}-containing medium. Figure shows changes in mean Fura-2 fluorescence intensity ratio (F340/380) in the absence and presence of Ver plus LOE (n = 2). Arrows indicate the administration of each drug.

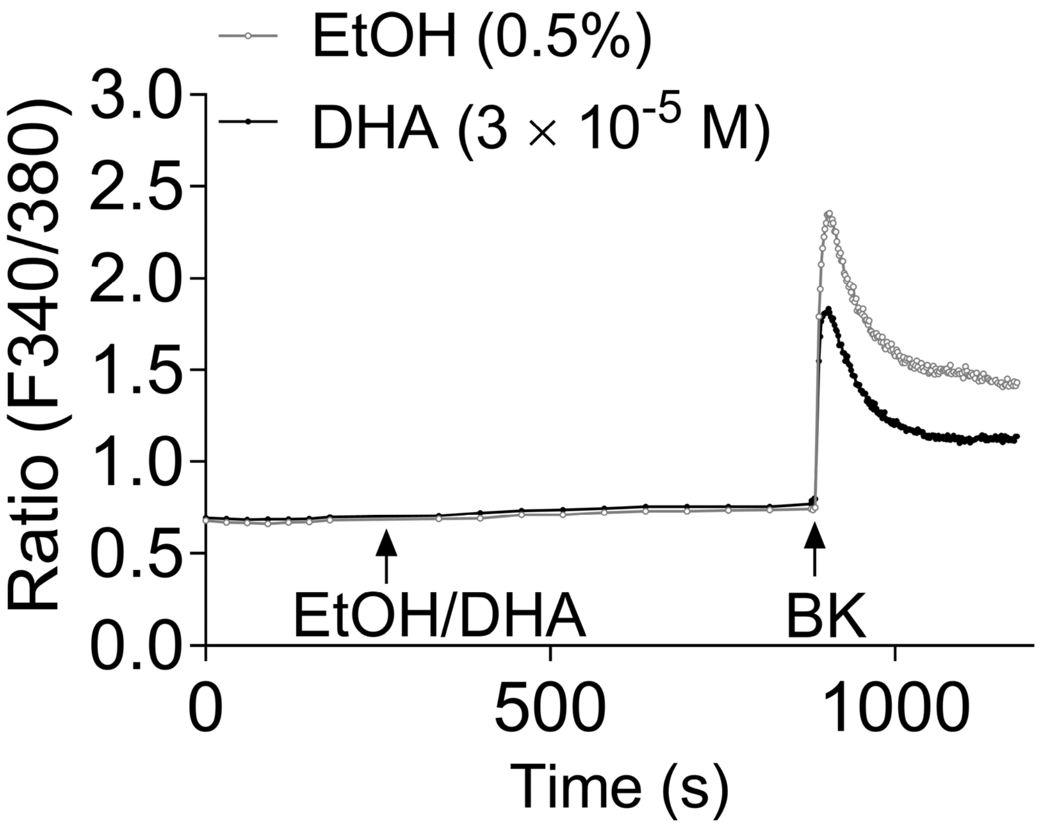

Supplementary Fig. 5

Effects of docosahexaenoic acid (DHA, 3×10^{-5} M) on intracellular Ca^{2+} increase induced by bradykinin (BK, 10^{-8} M) in B_2-293T cells in Ca^{2+}-containing medium. Figure shows changes in mean Fura-2 fluorescence intensity ratio (F340/380) in the absence and presence of DHA (each n = 3). Arrows indicate the administration of each drug. EtOH: ethanol.

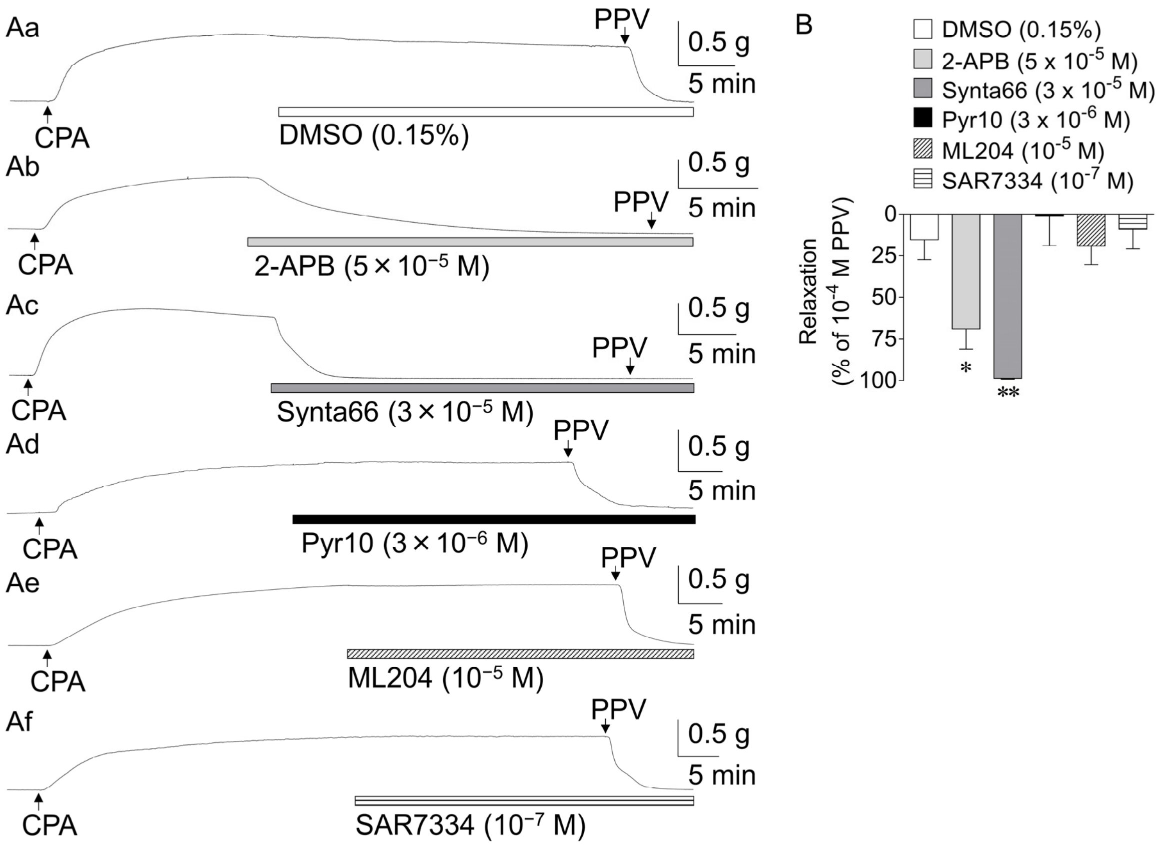

Supplementary Fig. 6

Representative traces (A) and summarized data (B) of the inhibitory actions of 2-aminoethoxydiphenyl borate (2-APB, a non-selective Orai/TRPC channel inhibitor, 5×10^{-5} M; b), Synta66 (a selective Orai1 inhibitor, 3×10^{-5} M; c), Pyr10 (a selective TRPC3 channel inhibitor, 3×10^{-6} M; d), ML204 (a selective TRPC4 channel inhibitor, 10^{-5} M; e), SAR7334 (a selective TRPC6 channel inhibitor, 10^{-7} M; f), and their vehicle (0.15% DMSO; a) on the contractions induced by cyclopiazonic acid (CPA, 3×10^{-5} M) in the presence of verapamil (10^{-5} M) plus LOE-908 (3×10^{-5} M) in guinea pig gastric fundus smooth muscle. Data are expressed as means ± standard error of the mean (n = 4–5). *$P < 0.05$, **$P < 0.01$ *vs.* DMSO (one-way ANOVA followed by Dunnett's test). EtOH: ethanol (0.1%), PPV: papaverine (10^{-4} M), DMSO: dimethyl sulfoxide.

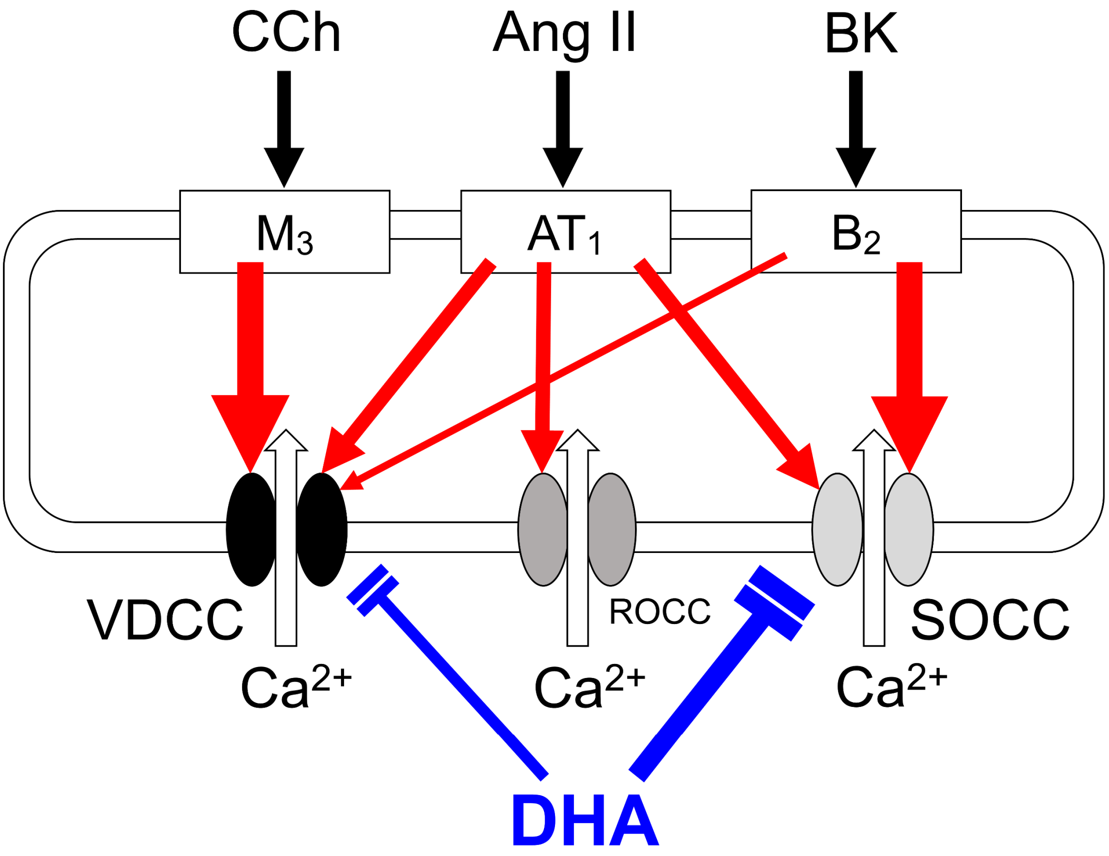

Supplementary Fig. 7

Graphical abstract of this study. Carbachol (CCh) contracts guinea pig (GP) gastric fundus smooth muscle (GFSM) by stimulating the acetylcholine M_3 receptor. This contraction almost completely depends on voltage-dependent Ca^{2+} channels (VDCCs). Angiotensin II (Ang II) contracts GP GFSM by stimulating the Ang II AT_1 receptor. This contraction depends on receptor-operated Ca^{2+} channels (ROCCs) and store-operated Ca^{2+} channels (SOCCs) in addition to VDCCs. Bradykinin (BK) contracts GP GFSM by stimulating the BK B_2 receptor. This contraction strongly depends on SOCCs, with a low contribution of VDCCs. We previously reported that docosahexaenoic acid (DHA) partly inhibits Ca^{2+} influx through VDCCs. In addition, we now show that inhibition of SOCC-mediated Ca^{2+} influx is a new mechanism responsible for the DHA-induced inhibition of GFSM contractions, which may be largely responsible for the inhibition of BK-induced contraction.

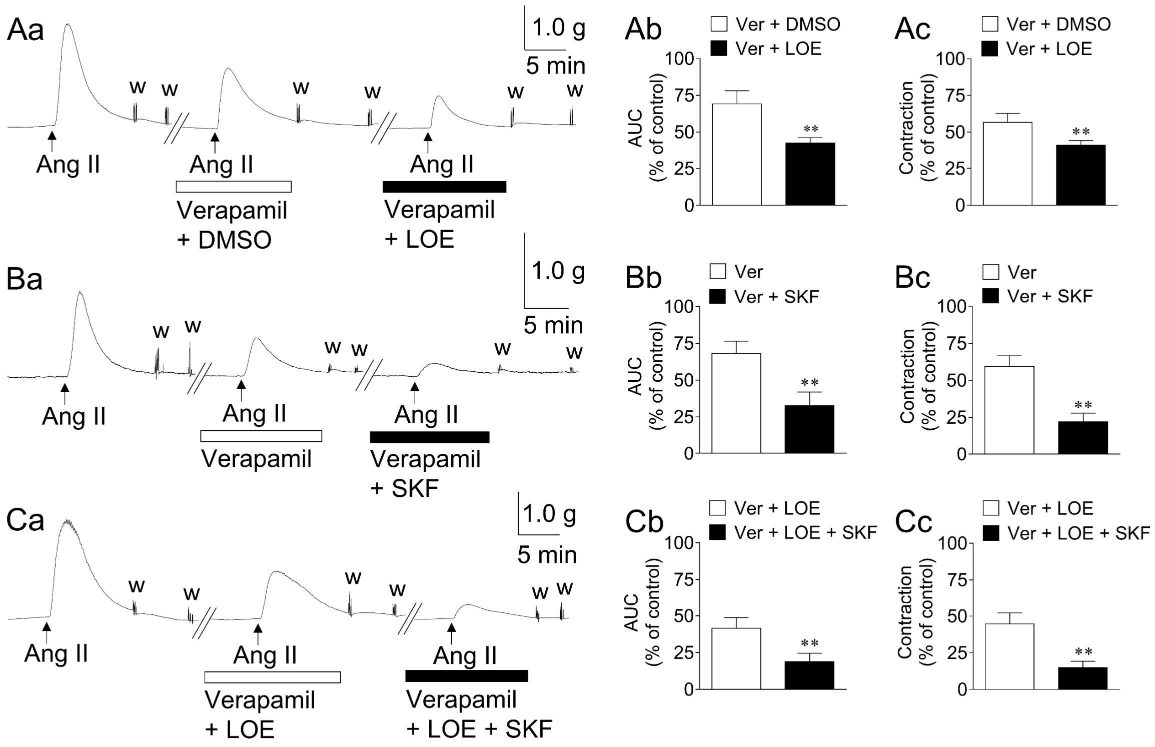

Supplementary Fig. 8

Representative traces (a) and summarized data (b: area under the curve (AUC); c: maximum contraction) of the effects of LOE-908 (LOE, 3 × 10^{-5} M; A) and SKF-96365 (SKF, 3 × 10^{-5} M; B, C) on the contractions induced by angiotensin II (Ang II, 10^{-6} M) in the presence of verapamil (Ver, 10^{-5} M; A, B) or Ver plus LOE (C) in guinea pig gastric fundus smooth muscle. Data are expressed as means ± standard error of the mean (n = 11 (A), n = 5 (B), and n = 7 (C)). **$P < 0.01$ *vs.* Ver plus DMSO/Ver/Ver plus LOE (paired *t*-tests). DMSO: dimethyl sulfoxide (0.015%), w: wash out.
